# Editorial: Drug Development and Target Discovery in Pulmonary Vascular Diseases

**DOI:** 10.3389/fphar.2020.00660

**Published:** 2020-05-08

**Authors:** Rui Zhang, Yang-Yang He, Zhi-Cheng Jing

**Affiliations:** ^1^Department of Cardio-Pulmonary Circulation, Shanghai Pulmonary Hospital, Tongji University School of Medicine, Shanghai, China; ^2^Department of Cardiology, Peking Union Medical College Hospital, Chinese Academy of Medical Sciences & Peking Union Medical College, Beijing, China

**Keywords:** pulmonary vascular disease, pulmonary arterial hypertension, drug development, targeted drug, molecular machine

Pulmonary vascular disease is a broad category of disorders threatening millions of people in the world, often leading to abnormal blood flow between the lungs and heart. Despite multiple drugs interfering with prostacyclin, endothelin, nitric oxide pathways were available for pulmonary arterial hypertension (PAH) over the past 15 years, it still remained severe and complicated clinical situation (Humbert et al., 2019). The new prevention and treatment strategies for PAH are a major clinical issue as well as a scientific challenge that needed to be addressed urgently. Discovery in pathobiological and cellular mechanisms of pulmonary vascular remodeling will promote the progression of PAH.

We organized the Research Topic entitled “Drug Development and Target Discovery in Pulmonary Vascular Diseases” to discuss and explore new therapeutic targets and mechanism of drug action toward pulmonary vascular disease. The topic covers both basic scientific as well as clinical aspects, consisting 4 original research articles and 1 abstract. Our Research Topic has been well received by the readership of the journal with over 5,000 views and more than 1,000 article downloads.

The feature of PAH is involved in the reduction of prostacyclin synthase expression and disorder of prostacyclin metabolic pathway. The clinical uses of prostacyclin analogues mimic endogenous prostacyclin, which lead to vasodilation and improvement of vascular remodeling (Galiè et al., 2003; Galiè et al., 2016). As a member of the prostacyclin family, iloprost was used for severe PAH and acute right ventricular (RV) failure. The importance for RV physiological changes during acute iloprost inhalation is discussed in the paper of Li et al. By cardiovascular magnetic resonance imaging, authors showed that acute iloprost inhalation increased RV ejection fraction and RV stroke volume, as well as decreased RV volume in idiopathic PAH and connective tissues disease associated PAH. These observations suggested RV function improvement would be largely attribute to a reduction of afterload by iloprost. This knowledge further added the clinical pharmacological value of iloprost for treatment of PAH.

As a mainstay of contemporary treatment, prostacyclin analogues therapies have been recommended to improve heamodynamics and outcomes in short term trials. (Barst et al., 1996). Beraprost is another chemically stable and orally active prostacyclin analogue. However, their present action mechanisms do not explain the relationship between their receptor subtype and ion channels better. Fan et al. demonstrated beraprost was able to down-regulate the expression of pulmonary prostacyclin receptors (IP) and reduce the activity of O_2_ sensitive potassium ion channels, partly through binding to the prostaglandin receptor E4 (EP4) in both humans and rats. This finding highlights a different binding receptor among iloprost, treprostinil, and beraprost, suggesting a specific E4 agonist may be potential therapeutic values for PAH.

Pulmonary vascular remodeling in PAH not only characterized by different vascular cells accumulation in the pulmonary arterial wall, but also by an exaggerated perivascular infiltration of inflammatory cells (Rabinovitch et al., 2014; Voelkel et al., 2016). Therefore, the possible pathobiological mechanisms of the vascular remodeling and inflammation are need to elucidate urgently. Deng et al. described a selective alpha 7 nicotinic acetylcholine receptor (α7nAChR) agonist, PNU-282987, which attenuated progression of monocrotaline (MCT)-induced PAH by downregulating the NLRP3. This article emphasized crosstalk between inflammatory cells and pulmonary artery smooth muscle cells played an important role in the pathogenesis of PAH. The α7nAChR is a ligand-gated ion channel and proved high expression in resident macrophages. The activation of this receptor inhibits the production of inflammatory cytokines, thereby attenuating the local inflammatory response.

In recent years, multiple molecular mechanisms have been explored a lot and new targets may constitute the theoretical basis for future clinical research. A PAH animal model induced by MCT is helpful to study the pathogenesis of PAH as well as pre-clinical assessment of new therapies (Hill et al., 2017). By this animal model, Zhang et al. identified an active ingredient of Chinese herbal medicine radix glycyrrhizae, 18β-Glycyrrhetinic acid (18β-GA), which has protective effects against PAH. In a previous study, these authors showed that 18β-GA played a critical role in antioxidant effect and lung protective activity. The present study corroborated the hypothesis that 18β-GA could reduce oxidative stress condition, mainly down-regulating the expression of both nicotinamide adenine dinucleotide phosphate oxidase-2 (Nox2) and Nox4.

In summary, the above all contributions provide an overview on potential therapeutic targets and discovery of drugs for pulmonary vascular disease. We further advocated the novel scientific knowledge needed to transfer into healthcare interventions better.

## Author Contributions

All authors listed have made substantial, direct, and intellectual contribution to the work and approved it for publication.

## Funding

This work was supported by grants from the Beijing Natural Science Foundation (7181009), National Natural Science Foundation of China (81630003), 13th Five-Year Plan-Precision Medicine-Key Research and Development Program-Clinical Cohort of Rare Disease (2016YFC0901500), CAMS Innovation Fund for Medical Sciences (2016-I2M-1-002, 2017-I2M-BR-02, 2017PT32016).

## Conflict of Interest

The authors declare that the research was conducted in the absence of any commercial or financial relationships that could be construed as a potential conflict of interest.
